# NK Cells in Autoimmune Diseases: Protective or Pathogenic?

**DOI:** 10.3389/fimmu.2021.624687

**Published:** 2021-03-12

**Authors:** Meifang Liu, Shujuan Liang, Cai Zhang

**Affiliations:** ^1^Key Lab for Immunology in Universities of Shandong Province, School of Basic Medical Sciences, Weifang Medical University, Weifang, China; ^2^School of Pharmaceutical Sciences, Cheeloo College of Medicine, Institute of Immunopharmaceutical Sciences, Shandong University, Jinan, China

**Keywords:** local microenvironment, immune tolerance, NK subsets, NK cells, autoimmune diseases

## Abstract

Autoimmune diseases generally result from the loss of self-tolerance (i.e., failure of the immune system to distinguish self from non-self), and are characterized by autoantibody production and hyperactivation of T cells, which leads to damage of specific or multiple organs. Thus, autoimmune diseases can be classified as organ-specific or systemic. Genetic and environmental factors contribute to the development of autoimmunity. Recent studies have demonstrated the contribution of innate immunity to the onset of autoimmune diseases. Natural killer (NK) cells, which are key components of the innate immune system, have been implicated in the development of multiple autoimmune diseases such as systemic lupus erythematosus, type I diabetes mellitus, and autoimmune liver disease. However, NK cells have both protective and pathogenic roles in autoimmunity depending on the NK cell subset, microenvironment, and disease type or stage. In this work, we review the current knowledge of the varied roles of NK cell subsets in systemic and organic-specific autoimmune diseases and their clinical potential as therapeutic targets.

## Introduction

Autoimmune diseases generally result from the loss of self-tolerance (i.e., failure of the immune system to distinguish self from non-self), which leads to the production of autoantibodies and self-reactive lymphocytes that cause tissue damage (1, 2). Although most autoimmune diseases are relatively uncommon, they are associated with significant morbidity and mortality. Nearly 100 distinct autoimmune diseases have been identified to date; these are organ-specific (e.g., primary biliary cirrhosis [PBC]) or are characterized by systemic immune dysfunction involving multiple organs [e.g., systemic lupus erythematosus (SLE)] (3, 4). Despite significant advances in the diagnosis and treatment of autoimmune diseases, many details of their pathogenesis and etiology have yet to be elucidated.

Autoimmune diseases principally develop as a result of abnormal activation of T and B cells. However, there is increasing evidence that natural killer (NK) cells—which link innate and adaptive immunity—play an important role in their development, for example in SLE, type 1 diabetes mellitus (T1DM), and autoimmune liver disease (ALD) (5–7). NK cells are cytotoxic lymphocytes that are critical for the defense against infections and tumors (8). NK cell activation is governed by the integration of activating and inhibitory signals from cell surface receptors (9, 10); NK cells detect cells that are under stress as a result of infection or malignancy and rapidly respond by secreting cytotoxic granules or death receptor ligands. In addition to their direct cytotoxicity, NK cells exert immunoregulatory functions in innate and adaptive immune responses by producing various cytokines and chemokines such as interferon (IFN)-γ, tumor necrosis factor (TNF)-α, granulocyte-macrophage colony-stimulating factor (GM-CSF), and chemokine (C-C motif) ligand (CCL)5 (11–13). They also exhibit immunologic memory, which challenges the conventional distinction between innate and adaptive immunity (14–18). NK cells shape the adaptive immune response through secreted cytokines and chemokines or crosstalk with other immune cells such as T and B cells and dendritic cells (DCs). Thus, NK cell hyperactivation or dysfunction is associated with the pathogenesis of various inflammatory and autoimmune diseases. However, NK cells have both protective and pathogenic functions in these diseases (19, 20) depending on the NK cell subset, microenvironment, and disease type and developmental stage. In this review, we discuss recent research on the diversity of NK cells and their roles in autoimmune diseases.

## Biological Characteristics of NK Cells

**Figure 1:**
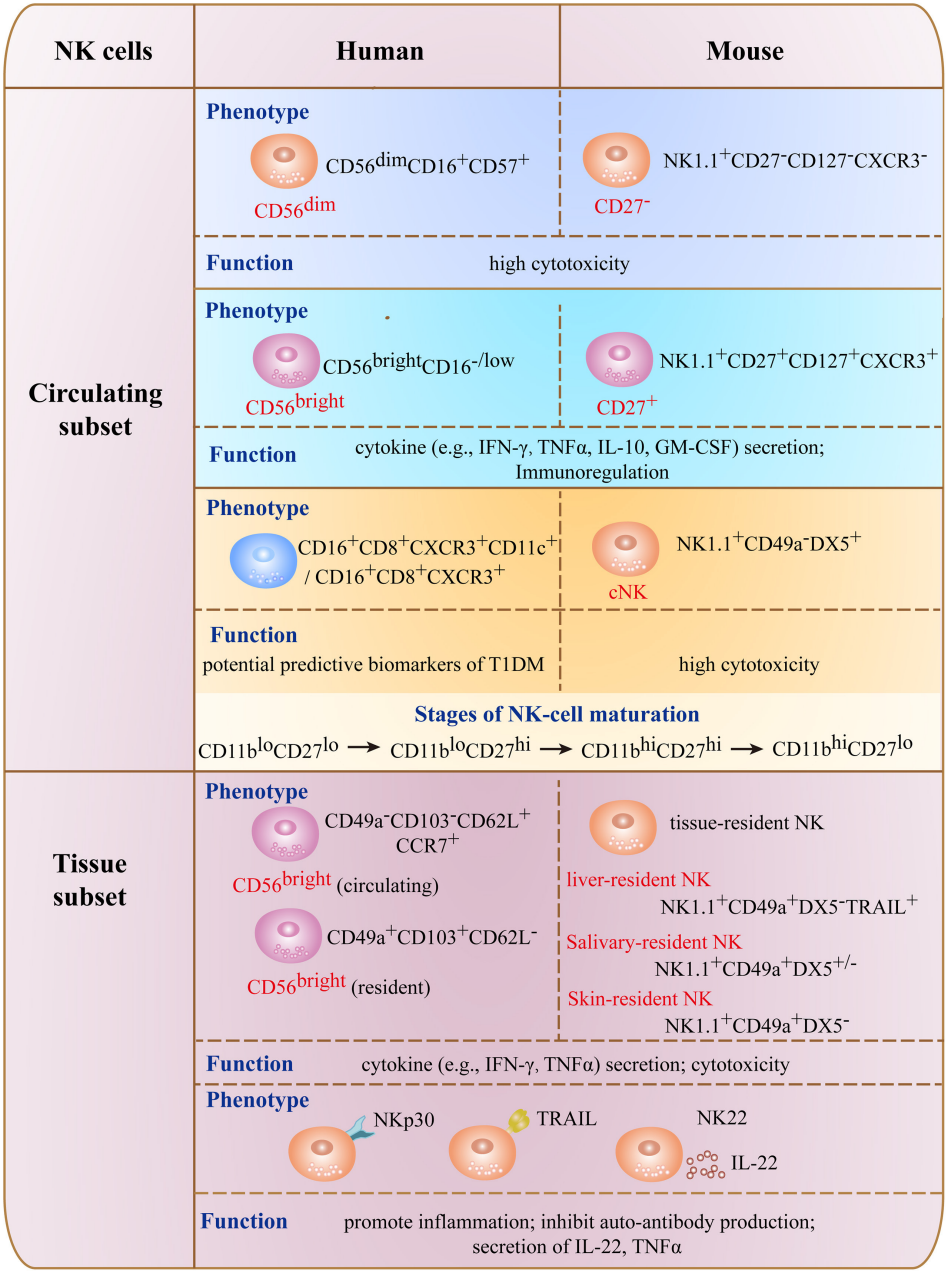
Phenotype and functions of major NK cell subsets in mouse and human.

NK cells are a heterogeneous population of innate lymphocytes comprising subsets with distinct phenotypes or cytokine secretion patterns (Figure 1) (20, 21). In humans, conventional (c)NK cells are divided into 2 major subsets based on the relative surface expression levels of cluster of differentiation (CD)56 and CD16 (FcγRIII). CD56^bright^CD16^−^ NK cells (also termed CD56^bright^ NK cells) are mostly present in secondary lymphoid tissues and produce cytokines such as IFN-γ, TNF-α, GM-CSF, interleukin (IL)-10, and IL-13 upon stimulation, thereby serving an immunoregulatory function in the maintenance of immune homeostasis (12, 13). Although CD56^bright^ NK cells have low cytotoxicity, this effect is enhanced under inflammatory conditions (12, 22). CD56^dim^CD16^+^ NK cells (also termed CD56^dim^ NK cells) exist predominantly in peripheral blood and express high levels of CD16; as they mature and become cytotoxic, they also express the terminal differentiation marker CD57 (23–25).

Circulating CD56^bright^ NK cells in peripheral blood are thought to be the precursors of CD56^dim^ NK cells (26). In murine NK cells lacking CD56 expression, CD27, CD127, and C-X-C motif chemokine receptor (CXCR)3 are important markers that distinguish NK cell subsets (27–29). CD27^−^, CD127^−^, and CXCR3^−^ NK cells have potent cytotoxicity resembling that of CD56^dim^ NK cells in humans, whereas NK cells positive for these markers are responsible for cytokine secretion and have low cytolytic capacity like the human CD56^bright^ subset.

There are 4 stages in the maturation of human and murine NK cells—i.e., CD11b^lo^CD27^lo^ → CD11b^lo^CD27^hi^ → CD11b^hi^CD27^hi^ → CD11b^hi^CD27^lo^ (30). Murine NK cells can also be divided into cNK and tissue-resident (tr)NK cells based on their production of IFN-γ and cytotoxicity (31–33). cNK cells are widely distributed and are CD49a^−^DX5^+^, whereas trNK cells have variable distribution in the uterus, skin, kidneys, salivary glands, and adipose tissue; the 2 cell types have common as well as unique features (31, 34–37).

Some factors in the tissue microenvironment (e.g., cytokines and ligands for NK cell receptors) contribute to differences in the composition of NK cell subsets and diversity of trNK cells. For example, liver-derived transforming growth factor (TGF)-β regulates and maintains the CD56^bright^ phenotype of human liver-resident NK cells by suppressing T-bet expression (38); and salivary gland-derived TGF-β directs the differentiation of salivary gland innate lymphoid cells including salivary-resident NK cells by inhibiting eomesodermin (Eomes) expression (34). Glomerulus-specific expression of NK group 2 member D (NKG2D) ligands was shown to be correlated with increased infiltration, maturation, and activation of NK cells in kidney during the development of lupus nephritis (39). NK cells can also be classified as NK1, NK2, or NK22 cells based on their cytokine secretion profiles (40, 41). With the development of detection methods such as mass cytometry, 2 NK cell subsets (CD16^+^CD8^+^CXCR3^+^ and CD16^+^CD8^+^CXCR3^+^CD11c^+^) were recently identified in patients at high risk for developing T1DM that can potentially serve as predictive biomarkers for the disease (42).

## NK Cells and Autoimmune Diseases

### NK Cells and Systemic Autoimmune Diseases

#### NK Cells and SLE

SLE is a progressive autoimmune disease with variable clinical manifestations affecting several organs including skin, lungs, blood, heart, and nervous system (43). It is characterized by the presence of nuclear autoantibodies along with abnormally activated T cells and hyperactive B cells, which form immune complexes that lead to inflammation (4, 44–46).

While adaptive immune mechanisms leading to organ damage in SLE have been extensively studied, the contribution of innate immune cells—especially NK cells—remains unclear. Several studies have provided evidence that NK cells are involved in SLE pathogenesis. For instance, the number of NK cells was markedly reduced in lpr mice, an SLE model; adoptive transfer of NK cells delayed the onset of autoimmunity, indicating a protective role for NK cells in SLE (47). It was also suggested that NK cells could delay SLE onset by suppressing autoantibody secretion by B cells. Recent studies have investigated the direct cytotoxic action and cytokine profiles of NK cells in the pathogenesis of SLE. For example, the increased cytotoxicity and proinflammatory phenotype of NK cells was shown to be correlated with downregulation of CD3ξ expression in patients with SLE (48); the authors also demonstrated that caspase-3 activity was higher in NK cells from patients with SLE than those from healthy controls and that this contributed to the downregulation of CD3ξ expression in the cells, which enhanced their proinflammatory phenotype.

NK cell subsets with different phenotypes, distributions, functions, and development have distinct roles in SLE. The proportion of NK cells (especially mature CD56^dim^ NK cells) was lower but that of CD56^bright^ NK cells was higher in the peripheral blood of SLE patients compared to healthy controls (49–51). It is worth noting that CD56^dim^ NK cells in active SLE patients showed increased IFN-γ production and an activated phenotype that included high expression of activating receptors (e.g., NKp44, NKp46, and CD69) and low expression of CD158a/h/g (51). The reduced number of circulating CD56^dim^ NK cells in SLE can be attributed to the migration of highly cytotoxic (CD56^dim^) NK cells from peripheral blood to kidneys in SLE patients and the consequent damage to local tissue. Many factors such as increased expression of NKG2D ligands [e.g., ribonucleic acid export [RAE]-1 and mouse UL16-binding protein-like transcript (MULT)-1], CD226 ligands (e.g., CD112 and CD115), proinflammatory cytokines (e.g., TNF-α), and chemoattractant chemokines [e.g., C-X3-C motif chemokine ligand (CX3CL)1] in kidney tissue potentially contribute to the migration of CD56^dim^ NK cells (39, 52–54). Similar NK cell migration was also observed in an animal SLE model, in which circulating DX5^+^ NK cells (mostly CD226^+^) infiltrated into the kidneys of MRL/lpr mice with an enhanced phenotype, which may be responsible for the kidney injury observed in SLE (52). Additionally, plasmocytoid (p)DC-secreted IFN-α mediated the activation-induced cell death of circulating NK cells in patients with active SLE, thereby contributing to the loss of circulating NK cells (52). A recent study showed that CD56^bright^ NK cells may contribute to SLE development. Serum IL-15 level was elevated in SLE patients, particularly those with active disease (55, 56); and an increased number of peripheral blood Ki67^+^CD56^bright^ NK cells was strongly correlated with elevated serum IL-15, clinical severity, and active nephritis in SLE patients (57). The high serum IL-15 level in SLE may be attributable to type I IFN-mediated DC activation; moreover, IL-15 induces the expression of Ki67 in NK cells, which stimulates NK cell proliferation and contributes to pathogenesis of SLE. However, the precise mechanisms by which CD56^bright^ NK cells promote SLE or mediate tissue injury remain unclear. It is thought that inflamed tissues recruit NK cells and alter their effector function by transforming their phenotype from one of low toxicity to that of cytotoxic CD56^bright^ NK cells via mechanisms that are as yet unknown (22). Thus, multiple factors in the local environment contribute to kidney infiltration by NK cells (especially CD56^dim^ NK cells) and tissue injury.

Most knowledge regarding the role of NK cells in SLE is derived from studies on peripheral blood; less is known about the functions of local NK cells in target tissues or trNK cells in SLE development. Recent studies using mouse SLE models have demonstrated a correlation between the number kidney-infiltrating NK cells and an active disease state (39, 52). The microenvironment of lupus nephritis promotes the maturation and maintenance of resident NK cells, as evidenced by a significantly larger CD11b^hi^CD27^lo^ NK cell fraction in the kidneys of SLE model mice (39). Notably, the kidney immune cell profile of SLE patients was recently established by single-cell RNA sequencing (58, 59); 21 subsets of leukocyte were identified including 2 clusters of trNK cells (CD56^bright^CD16^−^ and CD56^dim^CD16^+^) with both pro- and anti-inflammatory activities (58). Further studies are needed to determine the contribution of specific NK subsets to the pathogenesis of SLE.

#### NK Cells and Sjögren's Syndrome

SS is a chronic, multisystem disorder characterized by lymphocyte infiltration of target glands (e.g., lachrymal and salivary) and sicca symptoms (60, 61). SS can be divided into primary (p)SS or secondary SS depending on whether it occurs alone or in conjunction with other systemic autoimmune diseases such as SLE or rheumatoid arthritis (RA). The hallmarks of pSS are progressive focal infiltration of immune cells (mainly T and B cells), hypergammaglobulinemia, and the presence of autoantibodies, underscoring the importance of adaptive immunity in SS pathophysiology (62, 63). However, recent findings have suggested that innate immunity—especially NK cells—plays a role in SS pathogenesis (64–66). The relative and total numbers of circulating NK cells and CD56^dim^ NK cells were lower while the number of CD56^bright^ NK cells was higher in pSS patients compared to healthy control subjects (67, 68). The ratio of CD56^bright^ to CD56^dim^ NK cells was correlated with IgG level and showed diagnostic utility for pSS with good sensitivity and specificity (67). The lower numbers of circulating NK cells and CD56^dim^ NK cells in pSS patients may lead to their infiltration into glands and focal immune injury.

NK cells in salivary glands are predominantly tissue-resident; only a very small fraction is derived from peripheral blood (69). Salivary gland NK cells promote pSS progression by inducing IFN-γ production (70, 71). IL-33 expression is upregulated in the salivary glands of pSS patients and acts synergistically with IL-12 and IL-23 to stimulate IFN-γ secretion by NK cells, which contributes to pSS pathogenesis (72, 73). The overexpression of IL-12 in SS was also shown to participate in the differentiation of helper T (Th)1 cells and IFN-γ production (74, 75); and activation of IFN signaling and the recruitment of pDCs to the salivary glands of pSS patients may promote NK cell activation and IFN-γ production, thereby aggravating disease pathogenesis (71, 76, 77).

Different NK cell subsets in the salivary glands may play distinct roles in the pathogenesis of pSS. For example, NKp30^+^ NK cells are thought to accumulate in the minor salivary glands of pSS patients. The interaction of NKp30 with its ligand B7–H6 present on DCs and salivary gland epithelial cells induces IFN-γ secretion by NK cells, which enhances focal inflammation and cellular damage (78). NK22 cells—a subset of NK cells that produce IL-22—have also been detected in the salivary glands of pSS patients (79). Elevated IL-22 in the salivary glands acting synergistically with IL-17 and IL-23 plays a proinflammatory role in pSS (79, 80). On the other hand, NK22 cells secrete B cell-activating factor, which may contribute to B cell-mediated immunity in the development of pSS (81). Thus, NK cell subsets in the salivary gland have a pathogenic role in pSS. However, it was recently reported that a TNF-related apoptosis-inducing ligand (TRAIL)^+^ NK cells exerted a protective function in chronic infection-associated pSS by specifically eliminating activated CD4^+^ T cells in salivary glands (82). Taken together, most of the current data suggests a disease-promoting role for NK cells, while some NK cell subsets may have a protective function against pSS development.

#### NK Cells and Systemic Sclerosis

SSc, also known as scleroderma, is an immune-associated multisystem rheumatic disease characterized by vasculopathy, immune activation, and tissue fibrosis of skin and internal organs caused by abnormal production and deposition of collagen (83, 84). Although SSc is uncommon, affecting about 1 in 10,000 people worldwide, it has high morbidity and mortality (85). SSc can be divided into limited and diffuse cutaneous forms (lcSSc and dcSSc, respectively) (86).

The etiology and progression of SSc depend on multiple factors including immune activation (87, 88). Toll-like receptor (TLR)-mediated DC activation may be responsible for the variable immune responses observed in patients with different SSc phenotypes. Type I IFN-induced TLR expression by DCs may also contribute to the pathogenesis of SSc (89–91). However, the role of NK cells in SSc is not fully understood. Changes in the percentages, phenotype, and functions of peripheral blood NK cells have been observed in patients with SSc (92, 93); one study suggested that these changes were dependent on the SSc subtype, with increased NK cell numbers in dcSSc but not lcSSc (93). The activating receptor killer cell immunoglobulin-like receptor (KIR), two Ig domains and short cytoplasmic tail (KIR2DS)2 but not the corresponding inhibitory receptor was shown to be expressed on NK cells from SSc patients (94). Additionally, a linear increase in activated CD56^bright^ NK cells with SSc progression from early to definite SSc was demonstrated following TLR1/2 stimulation, highlighting the contribution of NK cells to SSc onset (95). NK cells secrete cytokines such as TNF-α, IL-6, and macrophage inflammatory protein (MIP)-1α and crosstalk with other immune cell types including DCs in response to TLR stimulation, thereby aggravating inflammation (96). Although the number of circulating NK cells is preserved in SSc, the cells show an unusual phenotype with decreased expression of chemokine receptors [CX3C chemokine receptor (CX3CR)1 and CXCR4], NKG2D, and CD69 (92). The reduced percentage of circulating CX3CR1^+^ NK cells in SSc patients may be due to their recruitment to target tissues in response to upregulation of CX3CL1 and NKG2D ligands in the inflamed endothelium. Moreover, NK cells from SSc patients induce endothelial cell activation, possibly exacerbating endothelial injury and contributing to SSc pathogenesis (92). A recent cytometry by time-of-flight study quantifying the proportions and phenotypes of circulating immune cells in patients with systemic autoimmune disease (including SSc) identified 12 cell populations that were altered compared to healthy controls, including a notable decrease in the size of the CD56^hi^ NK cell fraction (97). Thus, significant changes in immune cell populations occur in early-stage SSc, although how this contributes to disease onset remains to be determined. Most of the available evidence suggests a pathogenic role for NK cells in SSc that is related to disease stage and subtype (i.e., early or late stage of lcSSc and dcSSc).

#### NK Cells and Rheumatoid Arthritis (RA)

RA, one of the most prevalent chronic inflammatory diseases, is characterized by persistent synovitis, production of autoantibodies (especially against rheumatoid factor and citrullinated peptide), and cartilage and bone destruction, which lead to systemic complications including pulmonary, cardiovascular, skeletal, and psychological disorders (98, 99). The pathogenesis of RA is heterogeneous and complex and involves genetic and environmental factors, although the detailed mechanisms are not fully understood.

Genome-wide analyses have suggested that immune regulatory mechanisms underlie RA (100). RA results from the loss of immune self-tolerance, autoantigen presentation, and aberrant inflammatory cytokine production caused by abnormal activation of innate and adaptive immune systems (101). Although adaptive immunity (e.g., autoantibody production mediated by CD4^+^ T cells and B cells) predominates, innate immune cells (e.g., DCs, macrophages, mast cells, and NK cells) have also been implicated in RA pathogenesis (98, 102, 103).

Single-nucleotide polymorphisms in NK cell-related genes—especially *NKG2D* and MHC class I polypeptide-related sequence A (*MICA*)—were shown to be associated with RA susceptibility and severity (104–107). Early studies reported conflicting findings regarding the roles of NK cells in RA, which may be attributable to the fact that peripheral but not local synovial NK cells were examined; moreover, the different NK subsets were not distinguished, and the data were derived from different cohorts (108–110). The total numbers and percentages of peripheral NK cells were shown to be abnormally elevated in patients with active RA; this along with disease activity, autoantibody levels, and Th17/regulatory T cell (Treg) imbalance was correlated with increased serum IL-2 level (111). Higher percentages of peripheral NK cells were also found to be associated with elevated serum IL-15 level in RA patients compared to healthy controls. Circulating NK cells from RA patients showed decreased expression of the activating receptor NKp46 and higher expression of the inhibitory receptors CD158b and CD158e, which was associated with an impaired response to IL-15 (112). Decreased numbers of total peripheral NK cells and CD56^dim^ NK cells, but not the CD56^bright^ population, was observed in seropositive (i.e., positive for anticyclic citrullinated peptide antibodies and/or rheumatoid factor) patients with early RA (113). A decreased number of CD56^dim^ NK cells was associated with CD16-triggered NK cell apoptosis, and CD16 also triggered IFN-γ and TNF-α production by CD56^dim^ NK cells, thereby contributing to systemic inflammation (113).

Many studies have reported the accumulation of NK cells in the inflamed joints of RA patients, particularly in the active stage of the disease (114–116). The synovial CD56^bright^ NK cell population in inflamed joints expands and rapidly secretes IFN-γ in response to monocyte/macrophage-secreted IL-12, IL-15, and IL-18; IFN-γ activates macrophages and promotes TNF-α production (114, 115). The interaction between NK cells and monocytes/macrophages may constitute a positive feedback loop that leads to uncontrolled, persistent inflammation in RA. In addition to IFN-γ and TNF-α, GM-CSF plays an important role in RA (117, 118). Recently, synovial NK cells were shown to potentiate inflammatory arthritis through secretion of GM-CSF in mice (119). Synovial NK cells produce GM-CSF in an IL-18–dependent manner, which promotes neutrophil infiltration into inflamed joints and persistent arthritis. The suppressor of cytokine signaling (SOCS) family member cytokine-inducible SH2-containing protein (CIS) was identified as a direct negative regulator of GM-CSF signaling, suggesting that it can be a therapeutic target (119). Synovial fluid NK cells express high levels of the activating receptors NKG2D, DNAX accessory molecule (DNAM)-1, NKp44, and NKp46 and inhibitory receptor CD94/NKG2A, while synovial fibroblasts express multiple ligands for NK cell receptors. These activating receptors mediate the cytotoxic effects of NK cells on synovial fibroblasts, thus promoting local joint inflammation in RA (120). Synovial fluid NK cells also express high levels of C-C chemokine receptor (CCR)6, which promotes NK cell migration to the inflamed joint (121). An NK22 subset was identified in the synovial fluid of RA patients that plays an important role in the pathogenesis of RA by secreting IL-22 and TNF-α, which enhanced the proliferation of fibroblast-like synoviocytes (121, 122).

Most of the existing evidence indicates that synovial NK cells exert pathogenic effects in RA by directly promoting cell or tissue injury either by inducing cytotoxicity (e.g., in synovial fibroblasts) expressing activating ligands for NK cell receptors or by secreting inflammatory cytokines (e.g., IFN-γ, TNF-α, GM-CSF, and IL-22) that induce the activation of macrophages and neutrophils, thereby indirectly aggravating inflammation and tissue damage. The contribution of distinct NK subsets and their crosstalk with other cells in the inflammatory microenvironment at different stages of RA development await further study.

### NK Cells and Organ-Specific Autoimmune Diseases

#### NK Cells and ALD

ALD is a chronic liver disorder caused by the loss of tolerance to self-antigens in the liver. There are 3 types of ALD—namely, autoimmune hepatitis (AIH), primary sclerosing cholangitis (PSC), and PBC (123)—that have similar pathogenesis but different patterns of liver injury. AIH is characterized by infiltration of inflammatory cells around the portal tracts, which causes interface hepatitis. In PSC, the large hepatic bile ducts are targeted, leading to biliary tree obliteration, biliary cirrhosis, and portal hypertension. Damage to the small bile ducts in PBC results in portal tract destruction and biliary cirrhosis (123).

Adaptive immune responses are responsible for the progressive destruction of liver parenchyma in ALD. However, innate immune cells (especially NK cells) also contribute to ALD physiopathology (7). NK cells are enriched in the liver, accounting for up to 30–50% of total liver lymphocytes (124, 125). A direct role for NK cells in hepatocellular damage has been reported in AIH (126), and recent data from both animal models and clinical studies have provided further evidence for the contributions of different functional NK cell subsets in AIH. In a mouse model of polyinosinic:polycytidylic acid-induced hepatitis, which mimics human AIH histopathology, NK cells accumulated and were activated in the liver and caused hepatocyte injury, suggesting a pathogenic role for NK cells in AIH (127). In a concanavalin A-induced AIH model, IL-17C enhanced IL-2 expression in intrahepatic CD4^+^ T cells, which promoted NK cell activation and increased the number of liver NK cells and their activation; this was mostly due to a change in the CD3^−^NK1.1^+^NKP46^+^ NK cell fraction post treatment. There was also a significant upregulation of the surface markers CD25 and CD69 in liver NK cells, indicating that the change in the functional subset of NK cells in this model was induced by the cytokine environment (128). Additionally, murine CD49a^+^DX5^−^ lrNK cells were shown to play a pathogenic role during viral infection by inhibiting virus-specific T cell immunity in a programmed cell death protein (PD)-1/programmed death ligand (PD-L)1–dependent manner (129). Conversely, CD49a^−^DX5^+^ cNK cells—but not CD49a^+^DX5^−^ lrNK cells—in mice exerted a protective effect during acute infection with the hepatitis-B virus by promoting the antiviral activity of CD8^+^ T cells via IFN-γ secretion (130). Thus, distinct hepatic NK cell subsets (especially circulating and liver-resident populations) have different functions during AIH development. A clinical study found enhanced liver infiltration of NK cells in acute AIH patients, with higher numbers of CD56^bright^ but not CD56^dim^ NK cells before treatment that were markedly reduced after corticosteroid therapy. Infiltrated NK cells also expressed a high level CD161—a Th17 plasticity-associated marker—as well as the activation molecules perforin and granzyme B, and were also more resistant to Treg-mediated suppression, suggesting that inadequate regulation by exhausted Forkhead box (FOX)P3^+^ Tregs was responsible for the functional bias of NK cells and their pathogenic effects in AIH (131). A study combining clinical samples and experimental AIH demonstrated that a decrease in the size of the CD56^dim^ NK cell fraction in peripheral blood was negatively correlated with disease progression in AIH patients. In an animal model, hepatic accumulation of CXCR3^−^ cNK cells—equivalent to CD56^dim^ NK cells in humans—was accompanied by a reduction in the numbers of these cells in the periphery (blood, spleen, and bone marrow); this peripheral cNK cell type redistribution was associated with AIH progression (132). The same authors also observed that although the number of CD49a^+^ lrNK cells did not increase significantly, they showed an activation phenotype identical to cNK cells and contributed to AIH injury. These data imply that targeting NK cell activation and migration is a potential therapeutic strategy for AIH.

In patients with PBC, the number of cytotoxic NK cells was shown to be increased in peripheral blood and liver. These NK cells kill biliary epithelial cells in a TLR4-, TLR3-, or TRAIL-dependent manner (133–135). Given their expression of TRAIL, lrNK cells may be primarily responsible for PBC pathogenesis. Infiltration of NK cells into the liver was dependent on the C-X-C chemokine receptor (CXCR)1/C-X-C motif ligand (CXCL)8 axis and involved CD56^dim^ cells, which exerted cytotoxic effects against autologous biliary epithelial cells (136). In patients with PSC, the number of NK cells was increased in peripheral blood but not the liver (137, 138). The expression of ligands of inhibitory KIRs was significantly reduced in PSC patients (139), while that of ligands for activating NK cell receptors was upregulated (140, 141). These data strongly suggest that NK cells are activated and cytotoxic in PSC. A recent study reported higher proportions of CCR7^+^ NK cells in the peripheral blood and liver of PSC patients based on increased expression of the CCR7 ligand CCL21, suggesting that CCR7^+^ NK cell infiltration contributes to PSC pathogenesis (142). Data from the same study also showed a significantly higher number of CXCR3^+^ NK cells in peripheral blood but not in the liver of PSC patients. In contrast to the earlier view that NK cells play a pathogenic role, it was recently demonstrated that lrNK cells have an immunosuppressive function in the pathogenesis of PSC. In a dominant-negative TGF-β receptor (TGFβR)II mouse model that mimics key phenotypic features of human PBC, the progression of PSC was negatively correlated with lrNK cell counts. It was further demonstrated that DX5^−^CD11c^hi^ lrNK cells colocalized with and inhibited the proliferation of CD4^+^ T cells (143). These findings strongly suggest that liver-infiltrating NK cells participate in ALD development, although the detailed mechanisms require further investigation.

#### NK Cells and Multiple Sclerosis

MS is a chronic disease of the central nervous system (CNS) (144) that results from immune-mediated inflammation, demyelination, and subsequent axonal damage (145); it is characterized by progressive motor disability and cognitive deficits, and affects younger adults (146). The incidence of MS is increasing worldwide. Despite decades of research, the etiology of MS is still unknown. Increasing evidence points to the critical role of autoimmunity in MS development and progression (144, 147), with many studies focusing on the contribution of T and B cells (144, 147, 148). In fact, therapies targeting T and B cells have demonstrated clinical success in the treatment of MS; these are reviewed in detail elsewhere (149–151).

MS was previously considered as an organ-specific T cell-mediated autoimmune disease (152, 153). However, therapies targeting B cells have also been effective (154). Data from both MS patients and experimental autoimmune encephalomyelitis (EAE), an animal model of MS, indicate that NK cells are associated with disease activity and therapeutic response in MS (155, 156), although their precise role is controversial On one hand, blockade of the interaction between the NK cell inhibitory receptor NKG2A and its ligand Qa-1 with an antibody that is equivalent to human leukocyte antigen (HLA)-E alleviated CNS inflammation in EAE by promoting the cytotoxic action of NK cells on T cells and microglia (157, 158). Enrichment of CNS-resident NK cells by treatment with an IL-2/IL-2 antibody complex blocked MS progression by suppressing the myelin-reactive Th17 response (142). The same study also demonstrated that NK cells created a Th17-polarizing cytokine environment by increasing the levels of IL-1β, TGF-β, TNF, and proinflammatory cytokines including MCP-1 and MIP-1α; IL-2 complex treatment reversed this effect, highlighting its therapeutic potential for MS (142). MS disease severity was negatively correlated with the accumulation of NK cells in the CNS, which involved CX3CR1/CX3CL1 signaling (159). The number of peripheral CD3^−^CD56^+^ NK cells was higher IFN-β-treated RR-MS patients compared to untreated patients although NKG2D^+^CD3^−^CD56^+^ NK cell count and endogenous IL-22 level in CD3^−^CD56^+^ NK cells were lower in untreated RR-MS and CIS patients than in treated RR-MS patients, indicating that NK cells have a therapeutic role in MS while IFN-β treatment may direct them toward a proinflammatory phenotype (40). On the other hand, NK cells were shown to exacerbate EAE by promoting the expansion of M1 macrophages and encephalitogenic T cells into the CNS via secretion of IFN-γ (143). Through a mechanism involving KIR two Ig domains and long cytoplasmic tail (KIR2DL)4-HLA-G–mediated conjugation of human NK cells and oligodendrocytes (OLs), activated NK cells were polarized to express IFN-γ and exert cytotoxic effects against OLs, suggesting a mechanism by which NK cells promote MS pathogenesis (160). Data from MS patients and an EAE model also support a pathogenic role for NK cells in MS development. In EAE, contact with NK cells induced the release of IL-15 by neural stem cells (NSCs), which promoted NK cell proliferation and survival, thus contributing to CNS damage. Especially during the later stages of EAE, reduced surface expression of Qa-1 on NSCs resulted in their killing by NK cells; depleting NK cells during this phase alleviated disease severity (161). Most of these studies focused on total NK cells, whether different NK cell subsets play distinct roles in MS pathology remains an open question.

The major NK cell populations in the cerebrospinal fluid (CSF) and CNS are CD56^bright^ NK cells (162, 163). Along with a significant increase in total CD3^−^CD56^+^ NK cells, the regulatory CD56^bright^ NK cell fraction was also increased and persisted following alemtuzumab treatment (164). In relapsing–remitting (RR)-MS–the most common disease subtype—and clinically isolated syndrome, dysfunction of CD3^−^CD16^+^CD56^dim^ (CD56^dim^) and CD3^−^CD16^−^CD56^bright^ (CD56^bright^) NK cell subsets was shown to be associated with disease progression; specifically, the size of the latter cell population was significantly increased in IFN-β-treated RR-MS patients compared to untreated patients and healthy subjects (165). Another study demonstrated that a lower NK/CD4^+^ T cell ratio in IFN-β-treated early RR-MS patients had prognostic value for disease activity compared to the CD56^dim^ subset, while CD56^bright^ NK cell count was negatively correlated with CD4^+^ T cell and more specifically, CD4^+^IL-17A^+^ T cell numbers (166). These data indicate that CD56^bright^ NK cells have an immune regulatory function in MS that involves suppressing activated T cells and T cell subsets such as Th17 cells; moreover, CD56^dim^ NK cells appear to play a role in reducing disease activity (166). Interestingly, recent data from trials of various MS drugs such as IFN-β, fingolimod, and daclizumab indicate that the size of NK cell populations—specifically CD56^bright^ cells—is increased upon treatment (167, 168). A mass cytometry-based investigation of circulating CD56^bright^ cells in patients with RR-MS treated with daclizumab beta revealed an upregulation of CD56 in total NK cells along with multiple phenotypic changes in the CD56^bright^ cell population (169). One study examining the roles and biological features of CD27^high^ and CD27^low/−^ NK cells during the pre-disease onset stage in EAE found that the numbers of CD27^low/−^ NK cells in the spleen, lymph nodes, and bone marrow were increased whereas the number of CD27^high^ cells was decreased; this was accompanied by enhanced cytotoxicity of CD27^low/−^ NK cells and reduced IFN-γ production of CD27^high^ NK cells. Adoptive transfer of CD27^low/−^ NK cells aggravated EAE; and CD27^high^ but not CD27^low/−^ cells inhibited CD4^+^ T cell proliferation and Th17 cell differentiation. Moreover, these 2 subsets exhibited distinct roles in inducing the maturation of antigen-presenting bone marrow-derived macrophages and DCs. Thus, CD27^+^ NK cell subsets have different functions at the early stage of MS (170). Regulating CD56^+^ NK cell subsets is a potential therapeutic strategy for preventing MS onset.

NK cells contribute to MS pathogenesis by causing direct damage to CNS components (e.g., OLs) or exerting regulatory effects on autologous T cells (Th17 cells) by modulating the expression of activating and inhibitory receptors or secreting cytokines (IFNγ, IL-15, etc). Whether the functions of NK cells in MS are beneficial or detrimental is an open question, as most studies were conducted on peripheral NK cells rather than local tissue-resident or accumulated NK cells and have yielded conflicting findings. Novel experimental approaches are needed to evaluate the complex dynamics of NK cell subsets during MS development and their potential as therapeutic targets.

#### NK Cells and T1DM

T1DM is a typical organ-specific disease characterized by immune-mediated destruction of insulin-producing β cells in pancreatic islets (171), resulting in the loss of control of blood glucose levels even with insulin replacement therapy; this can in turn lead to ketoacidosis, severe hypoglycemia, and secondary complications (172). The innate and adaptive immune responses are involved in the destruction of pancreatic β cells (173–176), and recent studies have focused on the possible roles of NK cells in the initiation and progression of T1DM (177–180).

Evidence for the contribution of NK cells to the pathogenesis of T1DM has come from experimental models [e.g., BioBreeding diabetes-prone rat and non-obese diabetic (NOD) mouse] and clinical studies. NK cells are among the first immune cells to migrate to pancreatic islets (181, 182) and are involved in all stages of T1DM (183–185). The destructive effect of NK cells on pancreatic β cells was demonstrated by the decreased inflammation of pancreatic islets and T1DM remission induced by NK cell depletion (181, 186). Pancreatic NK cells of NOD mice with an inflammatory phenotype were found to express high levels of CD69 and CD25 and a low level of CD62 ligand (CD62L), suggesting their involvement in the development of T1DM (187). NK cells were also shown to contribute to the elimination of pancreatic β cells in an NKp46-dependent manner, leading to T1DM development (182). In NOD mice expressing an impaired NKG2D receptor in peripheral NK cells, exposure of pancreatic islet cells to the NKG2D ligand RAE-1 resulted in the downregulation of NKG2D and reduced NK cell cytotoxicity and IFN-γ secretion (188). NK cells in pancreatic islets were found to indirectly contribute to the destruction of pancreatic β cells by facilitating T cell activation (6, 189, 190). However, other studies have suggested that NK cells have a protective role in T1DM. For example, it was reported that NK cells limit the destruction of pancreatic β cells and contribute to T1DM remission by decreasing the numbers of autoreactive cytotoxic T cells (191); and a later study showed that increasing the proportion of NKG2D^+^ NK cells and inducing IFN-γ secretion in this population which enhancing the protective effect of complete Freund's adjuvant (CFA) in NOD mice (192). The mechanism underlying the modulation of NK cell function in the CFA treatment model is not clear, because the reason for the increased proportion of NK cells in the periphery and whether this influences the infiltration or functional status of pancreatic NK cells are unknown. NK1.1^+^/c-Kit^+^ NK cells were identified in the spleen, lymph, and islets of NOD mice that exerted direct cytolytic activity against activated insulin-specific CD8^+^ T cells in a PD-1/PD-L1–dependent manner (193). Adoptive transfer of these NK cells delayed diabetes development, suggesting an immunoregulatory and protective role for NK cells in T1DM (179, 193, 194).

Clinical data on the functions of NK cells in T1DM patients are scarce and ambiguous (179, 180, 195). Nonetheless, NK cells are known to play an important role in the immunopathogenesis of T1DM (196). A reduced number and decreased cytolytic activity of NK cells along with downregulation of activating receptors (e.g., NKG2D, NKp46, NKp30) or increased expression of inhibitory receptors (e.g., KIR2DL3) in peripheral blood mononuclear cells have been reported in T1DM patients (182, 195, 197–200), highlighting an association between NK cells and T1DM disease stage or risk (42, 201). A decrease in cytolytic activity of NK cells toward group B coxsackievirus (CV-B)–infected pancreatic β cells contributed to the persistence of CV-B and triggered autoimmunity in T1DM patients (197, 202). However, other studies found no correlation between T1DM clinical status and abnormalities in the number or function of NK cells (203, 204). One report showed that NK cells were rarely detected even in heavily inflamed pancreatic islets of T1DM patients, suggesting that they are not required for the death of pancreatic β cells (205). A bias in functional NK cell subsets has been observed that may be correlated with the development and progression of T1DM; although the number of CD3^−^CD56^+^ NK cells is reduced in patients with longstanding T1DM, there were no differences in the ratio of CD56^high^/CD16^−^ and CD56^low^/CD16^+^ NK cells between patients with longstanding or recent-onset T1DM and controls (195). Newly diagnosed young T1DM patients had fewer NK cells and effector CD56^dim^CD16^+^ and CD56^dim^CD16^−^ NK cell subsets compared to controls; moreover, T1DM patients with diabetic ketoacidosis (DKA) had fewer CD56^bright^CD16^−^ regulatory NK cells compared to patients without DKA (200). Hence, the relative proportions of NK cell subsets may be altered depending on the degree of metabolic impairment. Two novel NK cell subsets (CD16^+^CD8^+^ and CD16^+^CD8^−^) were identified in children at high risk of T1DM using dimensional reduction and computational unsupervised clustering approaches; and further analyses revealed that CD16^+^CD8^+^ (as CD16^+^CD8^+^CXCR3^+^) and CD16^+^CD8^+^CXCR3^+^CD11c^+^ NK cells were more abundant in high-risk individuals. However, whether CD11c expression reflects the activation status of NK cells in autoimmunity is unclear. The inconsistent results obtained in different studies may be attributable to the use of NK cell samples from peripheral blood, the lack of human pancreas specimens and functional markers of specific NK subsets, and the limited knowledge of the translocation of NK cells from peripheral to local organs during T1DM development.

## Conclusions and Future Perspectives

**Figure 2:**
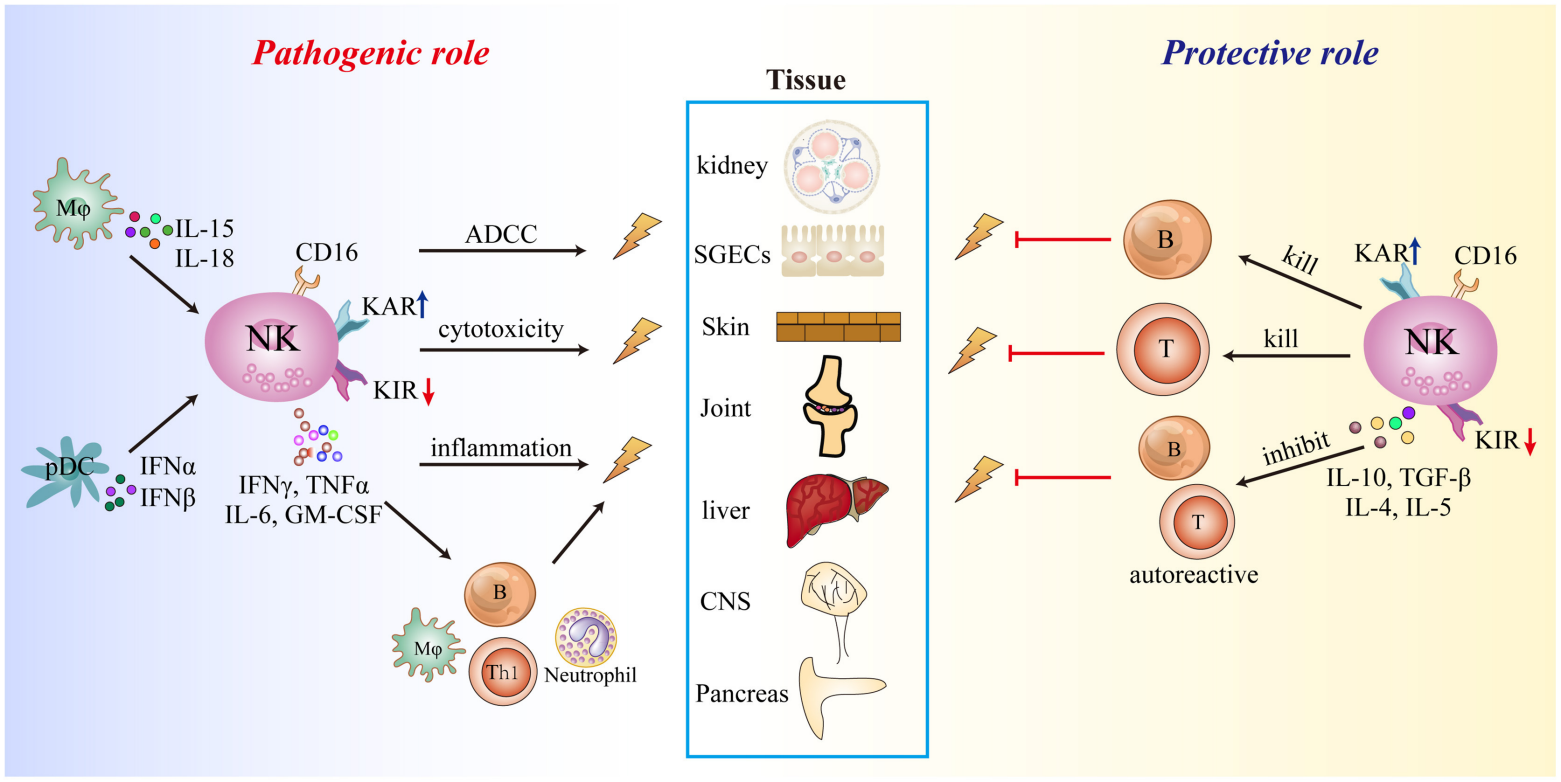
Summary of the role of NK cells in autoimmune diseases. KAR, killer cell-activating receptor; KIR, killer cell immunoglobulin-like inhibitory receptor.

The existing evidence demonstrates that NK cells are involved in the development of autoimmune diseases. The inflammatory microenvironment facilitates the migration of circulating NK cells into inflamed tissues and shapes the effector function of infiltrating and trNK cells, which involves upregulation of activating receptors and downregulation of inhibitory receptors, cell activation, and enhanced cytolytic activity and cytokine production (e.g., IFN-γ, TNF-α, and GM-CSF) leading to direct cell or tissue injury. NK cells can also bind autoantibodies through CD16 (FcγRIIIa) to mediate antibody-dependent cellular cytotoxicity. The cytokines secreted by NK cells recruit and activate other immune cells such as macrophages, neutrophils, and autoreactive T and B cells, thereby indirectly enhancing inflammation and tissue damage. However, some NK cells subsets may have a protective role in autoimmune diseases that involves direct killing of immature DCs and autoreactive T and B cells, or secretion of IL-10 to block the activation and effector functions of autoreactive T and B cells. The protective and pathogenic mechanisms of NK cells in autoimmune diseases are summarized in Figure 2.

There are many open questions regarding the precise functions and mechanisms of NK cells in autoimmune diseases. Based on current data, it is difficult to reconcile the contradictory roles (protective or pathogenic) of NK cells even in a single disease. Some early studies focused on bulk peripheral NK cells rather than specific NK cell subsets, especially local tissue-infiltrating or trNK cells. Single-cell RNA sequencing, mass spectrometry, and bioinformatics approaches have aided investigations of NK cell heterogeneity across different tissues (including diseased vs. healthy tissue) and the identification of distinct NK cell subsets involved in pro- and anti-inflammatory responses. The type of NK cell that accumulates in a tissue may determine the ultimate outcome of NK cell-mediated immune responses. Moreover, the tissue-specific microenvironment (e.g., cytokines, chemokines, ligands of NK cell receptors, and apoptosis-related molecules) and crosstalk between NK cells and other immune or stromal cells may shape the properties and functions of particular NK cell subsets. Notably, NK cells may have both beneficial or detrimental effects in the same autoimmune disease at different stages (e.g., onset, progression, relapse, or remission). Thus, future investigations should focus on tissue-specific NK cells and their interactions with other cells at the site of inflammation during different stages of disease development. Elucidating the precise roles of NK cells in disease initiation, progression, and resolution will provide insight into the pathogenic mechanisms of autoimmune diseases and that can guide the design of new therapeutic interventions.

## Author Contributions

ML, SL, and CZ were involved in the search and analysis of the literature, design and writing of the manuscript, and revision of the manuscript. All authors contributed to the article and approved the submitted version.

## Conflict of Interest

The authors declare that the research was conducted in the absence of any commercial or financial relationships that could be construed as a potential conflict of interest.
